# Homeodynamic complexity: multifractal analysis of physiological instability

**DOI:** 10.1186/cc10836

**Published:** 2012-03-20

**Authors:** A Ercole, SM Bishop, SI Yarham, VU Navapurkar, DK Menon

**Affiliations:** 1 University of Cambridge, UK

## Introduction

Physiological instability is a common clinical problem in the critically ill. Physiological adaptation can be regarded as a dynamic process, with stability being conferred by a number of apparently complex, fluctuating homeokinetic processes [1]. Many natural systems are nonlinear, and seemingly random fluctuations may result as a consequence of their underlying dynamics. Fractal geometry offers a method to characterize the underlying nonlinear state, providing a technique for monitoring complex physiology in real time, which may be of clinical importance.

## Methods

We employ the wavelet modulus maxima technique to characterize the multifractal properties of physiological time series such as heart rate (HR) and mean arterial pressure (MAP) under conditions of clinical physiological instability. We calculated point estimates for the dominant Hölder exponent (h_m_) and multifractal spectrum width-at-half-height (WHH). We investigated how these parameters changed with pharmacological interventions such as vasoconstriction.

## Results

**Figure 1:**
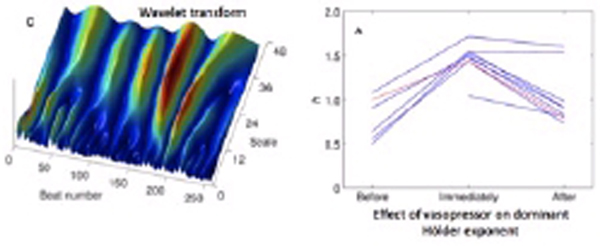


Hypotensive patients showed lower values of h_m _for MAP, consistent with a more highly fluctuating, antipersistent and complex behavior. Blood pressure support with pharmacological vasoconstriction led to a transient increase in h_m _for MAP (Figure 1) revealing the appearance of longer-range correlations, but did not affect h_m _as estimated for HR. On the other hand, supporting the heart rate with atropine had no effect on h_m _for MAP, but did tend to increase h_m _for HR.

## Conclusion

We demonstrate increasing signal complexity under physiological challenge consistent with the activation of homeokinetic processes. Differential fractal behavior for HR and MAP suggests that the homeokinetic systems are recruited in a targeted way depending on the physiological challenge. Pharmacological restoration of homeostasis leads to system decomplexification suggesting that homeokinetic mechanisms are derecruited as physiology is restored. We suggest fractal geometry provides a method for characterizing physiological instability and measuring the homeokinetic stress response during physiological challenges.

## References

[B1] Goldberger AL Amaral LAN Hausdorff JM Ivanov PC Peng C-K Stanley HE Proc Natl Acad Sci USA 2002 99 2466 2472 10.1073/pnas.012579499 11875196 PMC128562

